# Isolated thrombotic microangiopathy of the small intestine in a patient with atypical hemolytic uremic syndrome – a case report

**DOI:** 10.1186/s12882-020-01766-0

**Published:** 2020-03-24

**Authors:** Christoph Nunius, Maike Büttner-Herold, Simone Bertz, Mario Schiffer, Bjoern Buchholz

**Affiliations:** 1 Department of Nephrology and Hypertension, Friedrich-Alexander-University Erlangen-Nürnberg, Ulmenweg, 18 Erlangen, Germany; 2 Department of Nephropathology, Friedrich-Alexander-University Erlangen-Nürnberg, Erlangen, Germany; 3 Institute of Pathology, Friedrich-Alexander-University Erlangen-Nürnberg, Erlangen, Germany

**Keywords:** Thrombotic microangiopathy, Atypical hemolytic uremic syndrome, Small intestine, Renal transplant, Case report

## Abstract

**Background:**

Atypical hemolytic uremic syndrome (aHUS) is a rare disease characterized by systemic thrombotic microangiopathy (TMA) reflected by hemolysis, anemia, thrombocytopenia and systemic organ injury. The optimal management of aHUS-patients when undergoing kidney transplantation to prevent recurrence in the allograft is eculizumab, an approved recombinant antibody targeting human complement component C5.

**Case presentation:**

A 39 year-old woman presented with severe abdominal pain, diarrhea and emesis for 3 days. In her past medical history she had experienced an episode of aHUS leading to end stage renal disease (ESRD) in 2007 and a genetic workup revealed a heterozygous mutation in the *membrane cofactor protein* gene. In 2014 she underwent cadaveric kidney transplantation. Four years later she had to go back on hemodialysis due to allograft failure following a severe systemic cytomegalovirus infection resulting in transplant failure. At presentation she still received calcineurin-inhibitor therapy and reported subfebrile temperatures and pain projecting over the transplant prior to the current symptoms. A contrast enhanced CT-scan of the abdomen revealed inflammatory wall thickening of the small intestine. Diagnostic endoscopy discovered fresh blood in the small intestine without a clear source of bleeding. Histopathology of the small intestine biopsies showed severe thrombotic microangiopathy. Of note, the patient persistently had no signs of systemic hemolysis. Since the TMA of the small intestine was most likely due to aHUS, eculizumab treatment was initiated which abolished the symptoms.

**Conclusion:**

Here we report a patient with thrombotic microangiopathy with predominant manifestation in a single organ, the small intestine, due to aHUS with absence of systemic signs and symptoms. aHUS patients usually require a secondary trigger for the disease to manifest. In this case, the trigger may be attributed to the dysfunctional renal transplant, which was subsequently explanted. Histology of the explanted kidney showed severe inflammation due to purulent nephritis and signs of cellular rejection. After nephrectomy, we continued eculizumab therapy until the patient completely recovered. No signs of TMA recurred after discontinuation of eculizumab, further supporting the concept of the renal transplant as the main trigger of TMA of the small intestine in our patient.

## Background

Thrombotic microangiopathy (TMA) syndromes are defined by microangiopathic hemolytic anemia, thrombocytopenia and systemic organ injury [1]. TMA may be caused by atypical hemolytic uremic syndrome (aHUS), which usually can be referred to dysregulated activity of the alternate complement pathway. In at least 50% of the patients an inherited or acquired complement abnormality can be found [2]. aHUS is also known to affect the kidney, often resulting in renal insufficiency [3–5]. The optimal management of aHUS-patients, when undergoing kidney transplantation to prevent recurrence in the allograft, is eculizumab [2, 4, 6–9]. Eculizumab is an approved recombinant antibody against human complement component C5 with a high potency of inhibiting complement-mediated thrombotic microangiopathy [9–11].

We report the case of a 39 year-old woman who had an unusual recurrence of her known aHUS predominantly affecting the small intestine.

## Case presentation

**Fig. 1:**
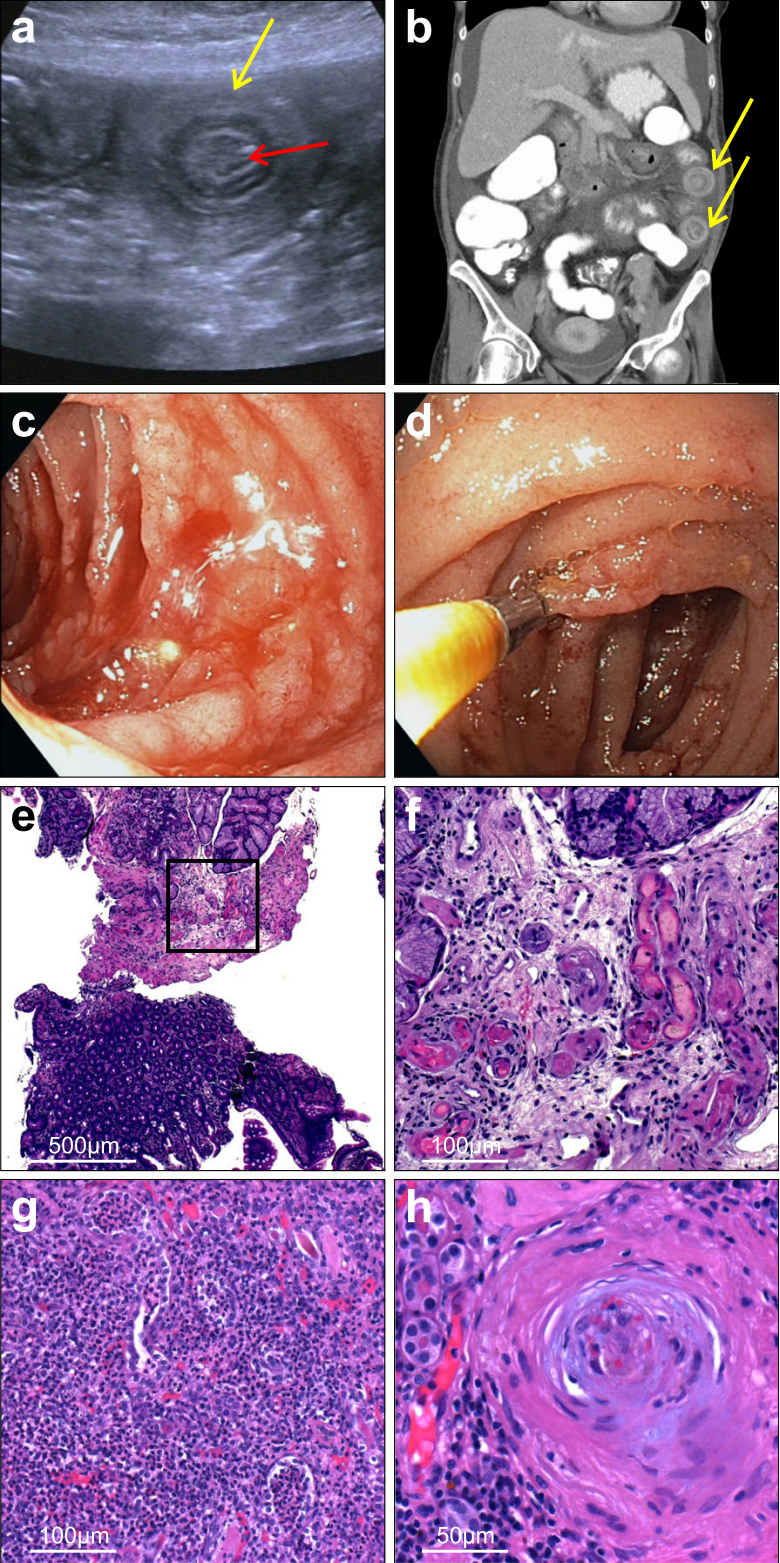
**a** Ultrasound of the small intestine showing dilated intestinal loop and wall thickening (yellow arrow) as well as signs of intussusception (red arrow). **b** Contrast-enhanced computer tomography of the abdomen showing extensive wall thickening of the small intestine (yellow arrows) and short-segment invaginations. **c** Esophagogastroduodenoscopy showing fresh blood in the small intestine with no clear source of bleeding. **d** Biopsy was taken from the small intestine. **e,f** HE staining of the duodenal biopsy showing thrombotic microangiopathy with fibrin insudation in the vascular wall, endothelial swelling and fresh microthrombi leading to lumen obliteration (f shows magnification of marked area in e). **g,h** HE staining of the kidney transplant showing severe granulocytic-purulent and lymphoplasmatic interstitial nephritis. **h** In addition, signs of preglomerular arterial thrombotic microangiopathy in an artery with “onion-skin” lesion and entrapped erythrocytes and fragmentocytes

The patient was a 39 year-old woman admitted to our ward because of severe abdominal pain, diarrhea and emesis for 3 days. No evidence of infectious gastroenteritis was found. She suffered from end-stage renal disease since 2007 because of aHUS due to a heterozygous disease-causing mutation resulting in a two base deletion with frameshift in the *membrane cofactor protein (MCP)* gene which leads to a STOP codon at AA 254. At the time she was treated with plasma exchange. In 2014 she underwent kidney transplantation. The kidney donor was a 58 year old male who died of myocardial infarction. The organ quality was considered good due to absence of any chronic alterations and achievement of primary function with a serum-creatinine of 1.2 mg/dL. In the following 2 years the patient suffered from progressively worsening transplant function which was assumed to be the consequence of a severe cytomegalovirus (CMV) infection with encephalitis and the need for nephrotoxic antiviral therapy. Unfortunately, no biopsy was performed to validate that assumption. Subsequently, in December 2018 she developed ESRD again and was treated with hemodialysis since then. The patient remained on the calcineurin inhibitor (CNI) tacrolimus as immunosuppressive therapy to avoid immunization since further kidney transplantation was planned. On admission an abdominal X-Ray showed no pathological findings, however an ultrasound suggested intussusception of the small intestine (Fig. 1a). Additionally, a contrast enhanced CT-scan of the abdomen showed inflammatory wall thickening of the small intestine (Fig. 1b). Consequently, a esophagogastroduodenoscopy (EGD) was performed, showing fresh blood in the small intestine with no clear source of bleeding (Fig. 1c,d).

Histopathological findings in the small intestine showed thrombotic microangiopathy with fibrin insudations in vascular walls, endothelial swelling and fresh microthrombi leading to lumen obliteration (Fig. 1e,f). The biopsy from the colon was normal. Both biopsies did not show any signs of cytomegalovirus infection or any other infectious agent.

The histological findings were rather surprising since the patient never had signs of systemic hemolysis reflected by normal bilirubin and haptoglobin values as well as absence of fragmented erythrocytes (Table 1). Solely, slightly elevated lactate dehydrogenase values could be found initially (Table 1). The patient’s ADAMTS13-levels were normal. There was no systemic sign of activation of the complement system (Table 1).

**Table 1:** Laboratory data on admission

Parameter	Patient Value	Reference
**ADAMTS13**		
ADAMTS-13-antibodies (U/mL)	1.8	< 12
ADAMTS-13-activity (IU/mL)	1.09	0.4–1.3
ADAMTS-13-antigen (IU/mL)	0.38	0.41–1.41
**Complement**		
Complement classical pathway (%)	0,79	74–151
Complement alternative pathway (%)	1,11	60–140
Complement C3 (mU/L)	16	< 40
sC5b-9 terminal Membrane attack complex (ng/mL)	184	58–239
**Hematology**		
Hemoglobin (g/dl)	11.3	11,8-15,5
Platetlet count (× 10^3^/μl)	290	160–400
White blood cell count (/μL)	6.92 × 10^3^	4–11
Schistocyte-Count	< 0.1	
**Blood chemistry**		
Lactate dehydrogenase (U/L)	565	< 250
Haptoglobin (mg/L)	309	300–2000
C-reactive protein (mg/L)	16.5	< 5
Total serum bilirubin (mg/dl)	0.4	< 1.1

Since TMA due to aHUS is often found to be triggered, we first stopped treatment with tacrolimus as a potential cause for TMA. Interestingly, during the last months the renal transplant caused pain accompanied by repeated subfebrile temperature. Therefore, inflammation due to renal infection or acute rejection was also considered as a potential trigger for TMA. Since TMA of the small intestine due to aHUS was highly probable, eculizumab treatment was initiated. Over the course of 5 days the patient’s condition improved rapidly. Another EGD was performed and a follow-up biopsy showed significantly improved histological findings. She could be discharged after 2 weeks of hospitalization without any complications and with continuation of the eculizumab therapy. However, since the kidney transplant was a probable cause of the disease, the patient was readmitted for a planned nephrectomy 2months later after complete recovery. No signs of recurrence of her thrombotic microangiopathy had occurred in the meantime.

After successful nephrectomy, histopathological findings were dominated by severe chronic tissue scarring with more than 90% interstitial fibrosis and tubular atrophy and severe inflammation with lymphoplasmacellular and granulocytic purulent interstitial nephritis and pyelitis as well as acute cellular rejection (Banff II). In addition, preglomerular arterial alterations were found going in line with previous and still mildly active thrombotic microangiopathy (Fig. 1g,h). Therefore, kidney pain and subfebrile temperatures prior to the first admission were likely due to severe renal inflammation.

## Discussion and conclusion

Here, we report a patient with an atypical course of aHUS.Our patient showed no systemic signs of aHUS: There was no evidence supporting hemolysis except for mildly elevated plasma lactate dehydrogenase. Plasma haptoglobin was normal, no fragmented erythrocytes were found. At no point did our patient show any signs of thrombocytopenia. Over a period of 2 weeks she suffered from anemia. However, this was not due to hemolysis but rather because of ESRD in concert with ongoing intestinal bleeding due to TMA of the small intestine. Therefore, we describe a non-systemic but almost exclusive affection of the small intestine by aHUS.

The patient had a known mutation in the *MCP* gene resulting in a premature STOP codon. Mutations in *MCP* are found in approximately 10% of aHUS patients [4]. *MCP* is a surface-bound complement regulatory protein which acts as a cofactor for Complement factor I-mediated cleavage of C3b and C4b deposited on host cells [4, 12]. This leads to an impaired protection of host cells from complement lysis [5]. As *MCP* is surface-bound, renal allografts will usually be able to correct the complement defect and therefore be protected against aHUS [4, 6]. Still, recurrence may happen in up to 20–50% of the cases and is possibly due to microchimerism in the graft or another unidentified mutation in a second complement regulatory protein [3, 4, 7, 13].

Patients with an underlying genetic mutation predisposing to aHUS usually require a secondary trigger for the disease to manifest [4]. Among these possible triggers are cerebral death, ischemia/reperfusion, infections and immunosuppressive drugs, including CNI [4, 8, 14]. Our patient was treated with CNI for years. CNI-induced aHUS typically manifests weeks after beginning therapy with CNI resulting in high plasma peak levels. Since CNI-induced TMA typically shows a dose-related course [1], it is rather unlikely that CNI therapy was the triggering event in our case. Nevertheless, it may be considered as a potential co-factor in our patient and therefore, CNI-based immunosuppressive regimen was discontinued. However, the main trigger may rather be attributed to the renal transplant and the severe inflammation due to infection and cellular rejection.

We opted not to treat the patient by plasma exchange but with eculizumab because of the underlying *MCP* mutation. *MCP* mutations are unlikely to respond to plasma exchange as *MCP* is not a plasma protein [3]. Eculizumab is highly effective in the treatment of aHUS as it targets C5 by blocking its cleavage to C5a and C5b. This way eculizumab inhibits the activation of the terminal complement pathway, which is essential for the development of the endothelial lesions characterizing aHUS [3]. There is evidence supporting the efficacy of eculizumab to induce remission of aHUS recurrence and being a viable prophylaxis to prevent post-transplant aHUS recurrence [6, 10, 15]. While normally *MCP* mutations pose a lower risk of recurrence, as described above, we decided to continue the eculizumab-based therapy as long as the renal transplant was still in situ. After nephrectomy, we continued eculizumab therapy for 3 months until the patient completely recovered and systemic inflammation was persistently absent. No signs of TMA recurred after discontinuation of eculizumab, further supporting the concept of the renal transplant as the trigger for TMA in our patient.

The patient expressed a strong desire to undergo another renal transplantation. Patients with aHUS should do this under protection of eculizumab [3, 4, 6, 8, 10] since transplantation and surgery may be triggers for TMA as well as CNI-therapy [7–9]. Therefore, we strongly suggest to apply eculizumab in case of another renal transplantation.

The optimal duration of eculizumab therapy after transplantation is still a matter of debate and remains elusive at the moment [6].

## Data Availability

The datasets used and/or analyzed during the current study are available from the corresponding author on reasonable request.

## Abbreviations

aHUS: Atypical hemolytical syndrome
TMA: Thrombotic microangiopathy
MCP: Membrane cofactor protein
CMV: Cytomegalovirus
ESRD: End-stage renal disease
CNI: Calcineurin inhibitor
EGD: Esophagogastroduodenoscopy
